# Mulligan Mobilization Combined with Conventional Therapy vs. Conventional Care Alone in Patients with Rotator Cuff Disease: A Systematic Review and Meta-Analysis of Randomized Controlled Trials

**DOI:** 10.3390/jcm14238352

**Published:** 2025-11-24

**Authors:** Abdulmuhsen Alqallaf, Abdullah M. Alharran, Plamen Penchev, Yousef Y. Alkandari, Bassam Almulla, Ahmed Almulla, Abdullah Alshatti, Abdulrahman Emad AlAyyaf, Ahmad Alahmad, Abdulrahman O. Al-Naseem

**Affiliations:** 1 Kuwait Institute for Medical Specializations, Kuwait City 12050, Kuwait; 2 Faculty of Medicine, Arabian Gulf University, Manama 329, Bahrain; 3 Faculty of Medicine, Medical University of Plovdiv, 4002 Plovdiv, Bulgaria; 4 AlRazi Orthopaedic Hospital, Kuwait City 12050, Kuwait; 5 Farwnia Hospital, Kuwait City 12050, Kuwait; 6 Department of Surgery, Royal College of Surgeons in Ireland, D09YD60 Dublin, Ireland; 7 Division of Orthopaedic Surgery, McGill University, Montreal, QC H3A 0G4, Canada

**Keywords:** rotator cuff disease, mulligan mobilization, conventional therapy

## Abstract

**Background/Objectives:** Rotator cuff disease (RCD) is one of the most common causes of shoulder dysfunction, often resulting in pain, limited range of motion (ROM), and reduced function. Mulligan Mobilization with Movement (MWM) has been proposed as an effective adjunct to conventional therapy by correcting positional faults and improving joint mechanics. However, the overall evidence in RCD remains inconclusive. This meta-analysis aimed to evaluate the efficacy of Mulligan mobilization combined with conventional therapy versus conventional therapy alone on pain, functionality, ROM, joint position sense, and quality of life (QoL) in patients with RCD. **Methods:** A comprehensive literature search was carried out in PubMed, Web of Science, Scopus, and the Cochrane Library from database inception to 12 October 2025, with no restrictions on publication year. We included randomized controlled trials (RCTs) that compared Mulligan mobilization combined with conventional therapy against conventional therapy alone in individuals with rotator cuff-related pain. The predefined outcomes were pain intensity, range of motion (ROM), quality of life (QoL), joint position sense, and functional performance. All statistical analyses were conducted using R version 4.3.1. Heterogeneity was assessed using the I^2^ statistic and the Cochrane Q test. Pooled mean differences (MDs) were calculated using the Inverse Variance approach with a restricted maximum-likelihood (REML) random-effects model. The review protocol was prospectively registered in PROSPERO (ID: CRD420251166854). **Results:** Four RCTs met the eligibility criteria and were included in the meta-analysis, comprising a total of 160 participants. Of these, 80 (50%) received Mulligan mobilization in combination with conventional therapy (mean age: 51 years; mean proportion of females: 45%). In the pooled analysis, Mulligan mobilization significantly improved pain at rest (MD −1.19; 95% CI [−1.64; −0.74]; *p* = 0.01; I^2^ = 0%), pain during activity (MD −2.25; 95% CI [−3.18; −1.31]; *p* = 0.01; I^2^ = 67%), functionality (MD −14.71; 95% CI [−20.10; −9.33]; *p* = 0.01; I^2^ = 51%), ROM (MD 19.92; 95% CI [11.25; 28.39]; *p* = 0.01; I^2^ = 58%), and joint position sense (MD −3.31; 95% CI [−6.22; −0.40]; *p* = 0.03; I^2^ = 80%) compared with conventional therapy alone. No significant difference was observed for QoL (MD 10.58; 95% CI [−3.18; 24.34]; *p* = 0.13; I^2^ = 76%). **Conclusions:** Mulligan mobilization combined with conventional therapy provides significant improvements in pain, functionality, ROM, and joint position sense in RCD. However, no statistically significant differences were observed in QoL between the groups. Integration of this technique into rehabilitation protocols may enhance clinical outcomes and functional recovery.

## 1. Introduction

Rotator cuff disease (RCD)—including tendinopathy, partial or full-thickness tears, and rotator cuff–related shoulder pain—is a leading cause of shoulder dysfunction, responsible for up to 70% of shoulder pain cases in adults [1,2]. Patients commonly experience persistent pain at rest and during activity, limited range of motion (ROM), impaired proprioception, and functional disability that significantly restricts daily activities and quality of life [2,3,4]. Conservative management remains the preferred first-line approach, typically incorporating exercise therapy, physical modalities, and manual therapy [2].

Mulligan Mobilization with Movement (MWM) is a manual therapy technique developed to correct subtle “positional faults” or joint “tracking errors” that may occur following injury or dysfunction [5]. The method combines a pain-free accessory glide applied by the therapist with simultaneous active movement by the patient, aiming to restore normal joint mechanics. MWM is reported to produce immediate reductions in pain and improved ROM through both biomechanical realignment and neurophysiological mechanisms, including activation of mechanoreceptors and descending pain inhibition [5,6].

Despite its increasing clinical use, evidence for Mulligan mobilization in RCD remains inconsistent. Individual randomized controlled trials (RCTs) have shown variable outcomes, largely due to differences in study design, intervention protocols, and sample sizes. Some reported significant improvements in pain and function [7,8], while others found no additional benefit beyond conventional physiotherapy [9,10]. Moreover, previous systematic reviews have often grouped heterogeneous shoulder conditions—such as impingement syndrome, adhesive capsulitis, and nonspecific shoulder pain—making it difficult to isolate the specific effects in RCT [11]. Existing literature also lacks a comprehensive meta-analysis evaluation. Previous meta-analyses have addressed the broader category of manual therapy for rotator cuff tendinopathy or focused on the general application of MWM across various shoulder conditions [12,13]. While highly valuable, these syntheses do not provide the necessary specificity for clinical decision-making regarding the exact protocol under investigation: Mulligan Mobilization combined with Conventional Therapy in patients with RCD. As a result, the certainty and generalizability of existing conclusions remain limited, resulting in no prior meta-analysis having exclusively synthesized evidence from RCTs focusing only on Mulligan Mobilization combined with conventional therapy in patients specifically diagnosed with RCD, leaving a knowledge gap in the literature. This gap underscores our focused review to provide a precise, high-level estimate of the effect size for this specific, widely used clinical protocol. Although the number of available RCTs is limited, a quantitative synthesis provides valuable pooled evidence for clinical guidance until larger multicenter trials are available. This meta-analysis, while encompassing limited evidence, provides the essential preliminary quantitative estimate of effect size necessary for clinical decision-making and for powering future large-scale trials.

This systematic review and meta-analysis addresses this literature gap by synthesizing data exclusively from RCTs evaluating Mulligan mobilization combined with conventional therapy versus conventional therapy alone in RCD. Using the GRADE framework and detailed subgroup and sensitivity analyses, this study aims to quantify the pooled effects on pain intensity, functionality, ROM, joint position sense, and QoL. The findings seek to clarify the true therapeutic impact of Mulligan techniques and provide evidence-based recommendations for their integration into routine physical therapy for rotator cuff–related shoulder dysfunction.

## 2. Methods

### 2.1. Inclusion/Exclusion Criteria

This systematic review and meta-analysis was conducted in accordance with the Cochrane Handbook for Systematic Reviews of Interventions and the Preferred Reporting Items for Systematic Reviews and Meta-Analyses (PRISMA) guidelines [14,15]. Ethical approval from an Institutional Review Board was not required because the analysis was based exclusively on previously published, publicly accessible data. Only peer-reviewed studies published in English were considered eligible, while gray literature was excluded. Studies were included in the meta-analysis if they satisfied all of the following criteria: (1) randomized controlled trials; (2) studies with patients with Rotator Cuff disease (participants were adults (≥18 years) clinically diagnosed with rotator cuff disease, including tendinopathy or partial/full-thickness tears confirmed by physical examination and/or imaging, with no prior shoulder surgery); (3) studies where patients underwent Mulligan mobilization combined with conventional therapy; (4) studies with a control group receiving conventional care (e.g., physiotherapy, exercise, or standard medical management); (5) studies that report at least one of the following outcomes: pain intensity at rest, pain intensity during activity, functionality, range of motion, quality of life (QoL), joint position sense assessed using validated measurement tools. Studies were excluded if any of the following conditions applied: (1) Studies not including patients with rotator cuff pathology; (2) studies in which Mulligan mobilization was not part of the intervention; (3) studies lacking a conventional care control group; (4) overlapping populations; (5) studies identified as gray literature (e.g., conference abstracts, dissertations) were excluded unless the full text was subsequently published as a peer-reviewed RCT that met all other eligibility criteria to ensure methodological rigor and data completeness; (6) no outcome of interest. This systematic review and meta-analysis was prospectively registered in PROSPERO (International Prospective Register of Systematic Reviews) under the identifier “CRD420251166854.”

### 2.2. Literature Search and Data Extraction

We systematically searched PubMed, Scopus, Web of Science and Cochrane Central from inception to 12 October 2025 with no year restrictions with the following search strategy: (“rotator cuff disease” OR “rotator cuff lesions” OR “rotator cuff syndrome” OR “Rotator Cuff Injuries” [Mesh]) AND (“mulligan mobilization” OR mulligan OR “mulligan technique” OR “mulligan concept” OR “mobilization with movement”) AND (“conventional care” OR “conventional exercise” OR “conventional physiotherapy” OR “conventional therapy”) AND (randomized controlled trial[pt] OR controlled clinical trial[pt] OR clinical trials as topic[mesh:noexp] OR trial[ti] OR random*[tiab] OR placebo*[tiab]). Backward citation tracking of the references of included studies was also performed to identify relevant studies beyond the database search. Two authors (P.P. and A.M.A.) independently extracted data using predefined criteria, including study characteristics, outcomes, and quality assessment methods, with the assistance of Rayyan software (https://new.rayyan.ai accessed on 13 October 2025) [16]. Discrepancies were resolved by discussion and consensus.

### 2.3. Endpoints and Subgroup Analyses

The meta-analysis assessed the following endpoints: pain intensity at rest, pain intensity during activity, functionality, range of motion, quality of life (QoL), and joint position sense. Subgroup analyses for each endpoint were conducted based on the risk of bias classification.

### 2.4. Quality Assessment

Risk of bias was evaluated using the Cochrane Collaboration’s tool for assessing risk of bias in randomized trials (ROBINS-II) [17], which categorizes studies as low risk, some concerns, or high risk of bias. Two authors (P.P. and A.M.A.) independently conducted the assessments, resolving disagreements by consensus. Publication bias was assessed using contour-enhanced funnel plots with the trim-and-fill method, which helps interpret asymmetry in relation to statistical significance thresholds, as recommended by Nakagawa et al. (2017) [18]. Other methods, such as p-curve or p-uniform analyses, were not feasible due to incomplete reporting of exact *p*-values or test statistics in the included studies. Following Cochrane guidelines, Egger’s test was not performed because fewer than 10 studies were included [14].

### 2.5. Statistical Analysis

For continuous outcomes, mean differences (MD) with 95% confidence intervals (CI) were calculated using the Inverse Variance method with a restricted maximum-likelihood estimator under a random-effects model [19,20]. Random-effects models were applied to account for demographic and methodological variability among studies. Heterogeneity was evaluated using the I^2^ statistic and the Cochran Q test. Two-sided *p*-values < 0.05 were considered statistically significant. Subgroup analyses based on risk of bias were conducted to minimize potential selection bias. Leave-one-out (LOO) sensitivity analyses were performed to assess the robustness of the results. A Baujat plot was generated to identify studies contributing most to heterogeneity and their influence on the overall meta-analytic results; this diagnostic tool visually depicts each study’s contribution to heterogeneity (*x*-axis) against its weight in the meta-analysis (*y*-axis), aiding interpretation of outliers or highly influential studies. All statistical analyses were performed using R version 4.3.1 with the “metafor” and “meta” packages [21]. In addition to MD, we calculated standardized effect sizes (Cohen’s d) for all continuous outcomes to facilitate clinical interpretability and enable comparison across measures. Because Cohen’s d may be positive or negative depending on the direction of the effect, magnitude was interpreted using absolute values. Conventional thresholds were applied, with absolute effect sizes of approximately 0.20, 0.50, and 0.80 considered small, moderate, and large, respectively [22].

## 3. Results

### 3.1. Study Selection and Baseline Characteristics

**Figure 1:**
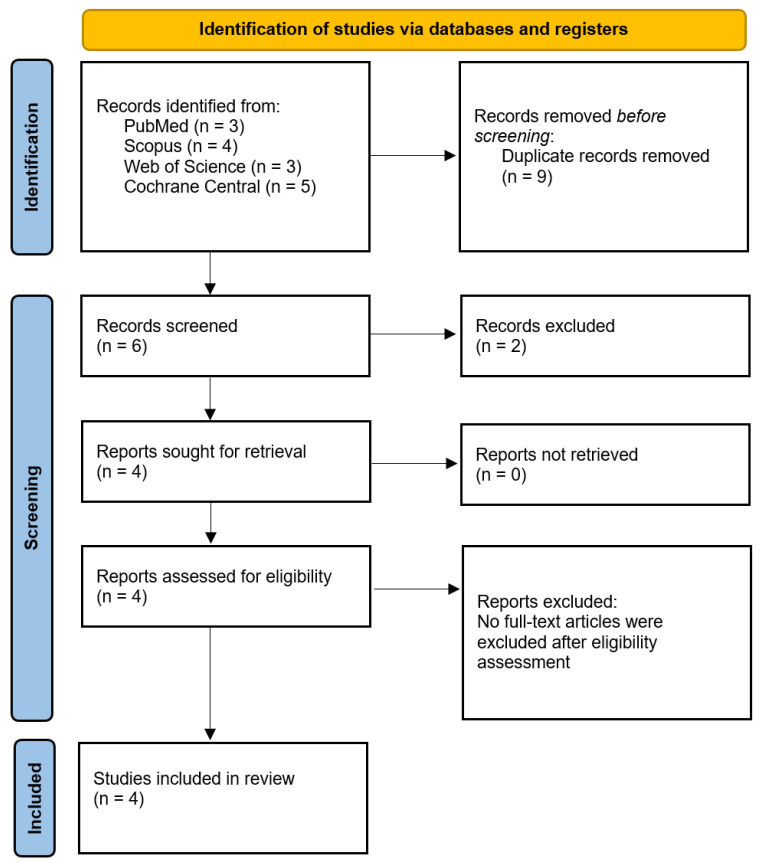
PRISMA flow diagram and study selection.

The search strategy yielded a total of 15 results. After removing duplicate records and unrelated articles or abstracts, a total of 4 full-text articles were assessed for eligibility, and all met the inclusion criteria; therefore, no full-text exclusions occurred, with 160 patients included [7,8,9,10]. Of these, 80 patients (50%) underwent Mulligan mobilization combined with conventional therapy and were included in our analyses. The mean age of the population was 51.7 ± SD years. The females accounted for a mean of 45%. It is noteworthy that all four eligible RCTs were conducted in Turkey. Population characteristics are presented in Table 1 and study contribution to meta-analysis in Table 2 and Figure 1.

### 3.2. Pooled Analyses of All Included Studies

#### 3.2.1. Pain Intensity at Rest

**Figure 2:**
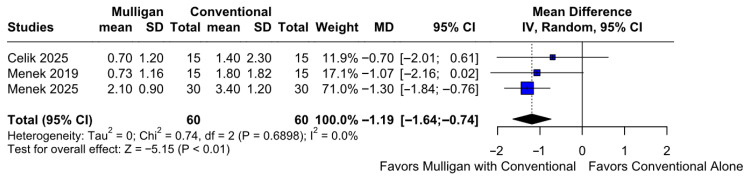
Forest plot illustrating the effect of Mulligan mobilization combined with conventional therapy versus conventional therapy alone on greater pain intensity at rest. The pooled results show a significant reduction in pain for the combined therapy group [7,9,10].

Mulligan mobilization combined with conventional therapy significantly reduced greater pain intensity at rest compared to conventional therapy only (MD −1.19; 95% CI [−1.64; −0.74]; *p* = 0.01; I^2^ = 0%) (Figure 2). This corresponded to a Cohen’s d of −0.80, reflecting a large effect, indicating a large magnitude of improvement following Mulligan mobilization (Supplementary Table S1). A LOO sensitivity analysis was conducted to assess the robustness of our findings. The overall effect size remained consistent across all iterations, with results remaining statistically significant in each case (MD −1.19; 95% CI [−1.64, −0.74]; *p* = 0.01; I^2^ = 0%) (Figure S1). This indicates that no single study had a disproportionate impact on the overall outcome. The Baujat plot suggested that the study by Menek et al. (2025) [10] was potentially influential, contributing substantially to the overall heterogeneity and effect size (Figure S2). According to the GRADE assessment, the certainty of evidence for this outcome was rated as high (Table 2).

#### 3.2.2. Pain Intensity During Activity

**Figure 3:**
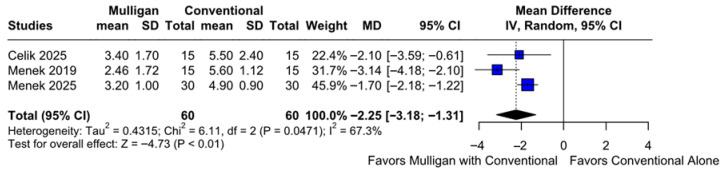
Forest plot illustrating the effect of Mulligan mobilization combined with conventional therapy versus conventional therapy alone on pain intensity during activity. The pooled results indicate a significantly greater reduction in pain for the combined therapy group [7,9,10].

A significantly greater reduction in pain intensity during activity was observed in patients treated with Mulligan mobilization and conventional therapy compared to conventional therapy only (MD −2.25; 95% CI [−3.18; −1.31]; *p* = 0.01; I^2^ = 67%) (Figure 3). This corresponded to a Cohen’s d of −1.60 reflecting a large effect, indicating a large magnitude of improvement following Mulligan mobilization (Supplementary Table S1). A LOO sensitivity analysis was performed to assess the robustness of our findings. The overall effect size remained consistent across all iterations, and the results were statistically significant in every case (MD −2.25; 95% CI [−3.18, −1.31]; *p* = 0.01; I^2^ = 67%) (Figure S3). This indicates that no single study had a disproportionate influence on the overall outcome. The Baujat plot highlighted the study by Menek et al. (2025) [10] as potentially influential, contributing substantially to the overall effect size, while Menek et al. (2019) [7] contributed notably to the overall heterogeneity (Figure S4). According to the GRADE assessment, the certainty of evidence for this outcome was rated as high (Table 2).

#### 3.2.3. Functionality

**Figure 4:**
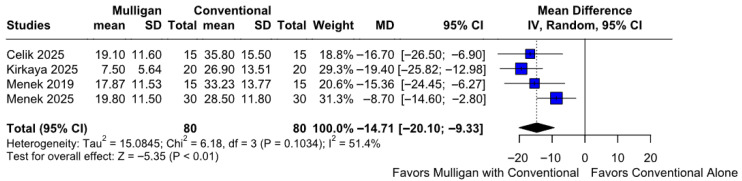
Forest plot illustrating the effect of Mulligan mobilization combined with conventional therapy versus conventional therapy alone on patient functionality. The combined therapy group experienced a significantly greater improvement in functionality [7,8,9,10].

Patients treated with Mulligan mobilization combined with conventional therapy significantly experienced better functionality compared to those treated with conventional therapy only (MD −14.71; 95% CI [−20.10; −9.33]; *p* = 0.01; I^2^ = 51%) (Figure 4). This corresponded to a Cohen’s d of −1.24, reflecting a large effect, indicating a large magnitude of improvement following Mulligan mobilization (Supplementary Table S1). A LOO analysis was performed to test the robustness of our results. The overall effect size remained consistent across all iterations, and the result remained significant in all cases (MD −14.71; 95% CI [−20.10; −9.33]; *p* = 0.01; I^2^ = 51%) (Figure S5). This suggests that no single study has a disproportional influence on the overall outcome. The Baujat plot identified in the studies by Menek 2025 et al. [10] and Kirkaya 2025 et al. [8] as potentially influential, contributing substantially to the overall result, and heterogeneity (Figure S6). According to the GRADE assessment, the certainty of evidence for this outcome was rated as high (Table 2).

#### 3.2.4. Range of Motion

**Figure 5:**
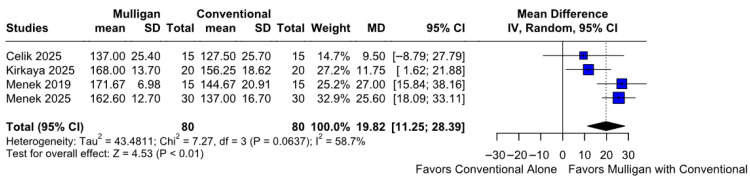
Forest plot illustrating the effect of Mulligan mobilization combined with conventional therapy versus conventional therapy alone on the range of motion. The combined therapy group achieved a significantly better range of motion [7,8,9,10].

Mulligan mobilization combined with conventional therapy provided significantly better range of motion compared to conventional therapy alone (MD 19.92; 95% CI [11.25; 28.39]; *p* = 0.01; I^2^ = 58%) (Figure 5). This corresponded to a Cohen’s d of 1.11, reflecting a large effect, indicating a large magnitude of improvement following Mulligan mobilization (Supplementary Table S1). A leave-one-out (LOO) sensitivity analysis was conducted to evaluate the robustness of our results. The overall effect size remained consistent across all iterations, with results remaining statistically significant in each case (MD −14.71; 95% CI [−20.10, −9.33]; *p* = 0.01; I^2^ = 51%) (Figure S5). This indicates that no single study had a disproportionate influence on the overall outcome. The Baujat plot identified the studies by Menek et al. (2025) [10] and Kirkaya et al. (2025) [8] as potentially influential, contributing substantially to both the overall effect size and heterogeneity (Figure S8). According to the GRADE assessment, the certainty of evidence for this outcome was rated as high (Supplementary Table S1).

#### 3.2.5. Join Position Sense

**Figure 6:**
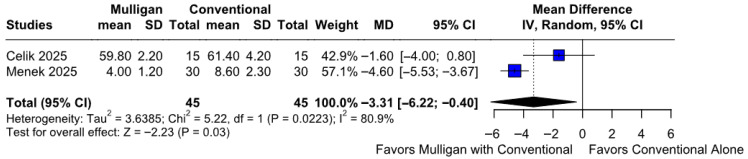
Forest plot illustrating the effect of Mulligan mobilization combined with conventional care versus conventional care only on joint position sense. The combined therapy group experienced a significantly better joint position sense [9,10].

Patients treated with Mulligan mobilization combined with conventional care experienced significantly better joint position sense compared to those treated with conventional care only (MD −3.31; 95% CI [−6.22; −0.40]; *p* = 0.03; I^2^ = 80%) (Figure 6). This corresponded to a Cohen’s d of −1.47, reflecting a large effect, indicating a large magnitude of improvement following Mulligan mobilization (Supplementary Table S1). A LOO sensitivity analysis was conducted to assess the robustness of our findings. The overall effect size remained consistent across all iterations, with results remaining statistically significant in every case (MD −3.31; 95% CI [−6.22, −0.40]; *p* = 0.03; I^2^ = 80%) (Figure S9). This indicates that no single study had a disproportionate impact on the overall outcome. The Baujat plot identified the study by Menek et al. (2025) [10] as potentially influential, contributing substantially to the overall effect size, while Celik et al. (2025) [9] contributed notably to the overall heterogeneity (Figure S10). According to the GRADE assessment, the certainty of evidence for this outcome was rated as high (Table 2).

#### 3.2.6. QoL

**Figure 7:**
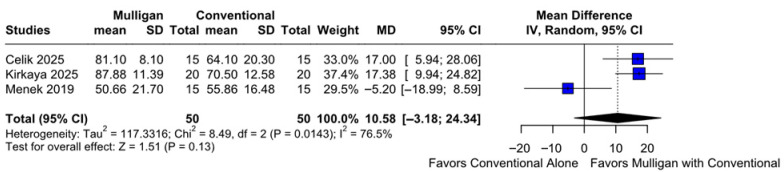
Forest plot comparing the effect of Mulligan mobilization combined with conventional therapy versus conventional therapy alone on QoL. No statistically significant difference was observed between the two groups, with high heterogeneity noted across studies [7,9,10].

There were no statistically significant differences between patients treated with Mulligan mobilization combined with conventional therapy and those treated with conventional therapy only (MD 10.58; 95% CI [−3.18; 24.34]; *p* = 0.13; I^2^ = 76%) (Figure 7). A LOO sensitivity analysis was conducted to assess the robustness of our findings. The overall effect size remained consistent across all iterations, with results remaining non-significant in every case (MD 10.58; 95% CI [−3.18, 24.34]; *p* = 0.13; I^2^ = 76%) (Figure S11). This indicates that no single study had a disproportionate influence on the overall outcome. The Baujat plot identified the study by Kirkaya et al. (2025) [8] as potentially influential, contributing substantially to the overall effect size, while Menek et al. (2019) [7] contributed notably to the overall heterogeneity (Figure S12). According to the GRADE assessment, the certainty of evidence for this outcome was rated as high (Table 2).

### 3.3. Subgroup Analyses

#### 3.3.1. Pain Intensity at Rest Based on the ROB

No statistically significant differences between the subgroups were observed (MD −1.19; 95% CI [−1.64; −0.74]; *p* = 0.4364; I^2^ = 0%) (Figure S13).

#### 3.3.2. Pain Intensity During Activity Based on the ROB

No statistically significant differences were observed between the subgroups (MD −2.25; 95% CI [−3.18; −1.31]; *p* = 0.8156; I^2^ = 67%) This corresponded to a Cohen’s d of −0.80 reflecting a large effect (Figure S14).

#### 3.3.3. Functionality Based on the ROB

No statistically significant differences were observed between the subgroups (MD −14.71; 95% CI [−20.10; −9.33]; *p* = 0.6925; I^2^ = 51%) This corresponded to a Cohen’s d of −0.80 reflecting a large effect (Figure S15).

#### 3.3.4. Range of Motion Based on the ROB

No statistically significant differences were observed between the subgroups (MD −19.82; 95% CI [11.25; 28.39]; *p* = 0.2476; I^2^ = 58%) (Figure S16).

#### 3.3.5. Joint Position Sense Based on the ROB

A statistically significant difference between the subgroups was observed (MD −3.31; 95% CI [−6.22; −0.40]; *p* = 0.0223; I^2^ = 80%) This corresponded to a Cohen’s d of −1.47 reflecting a large effect (Figure S17).

#### 3.3.6. QoL

No statistically significant differences were observed between the subgroups (MD 10.58; 95% CI [−3.18; 24.34]; *p* = 0.4212; I^2^ = 76%) (Figure S18).

### 3.4. Quality Assessment

**Figure 8:**
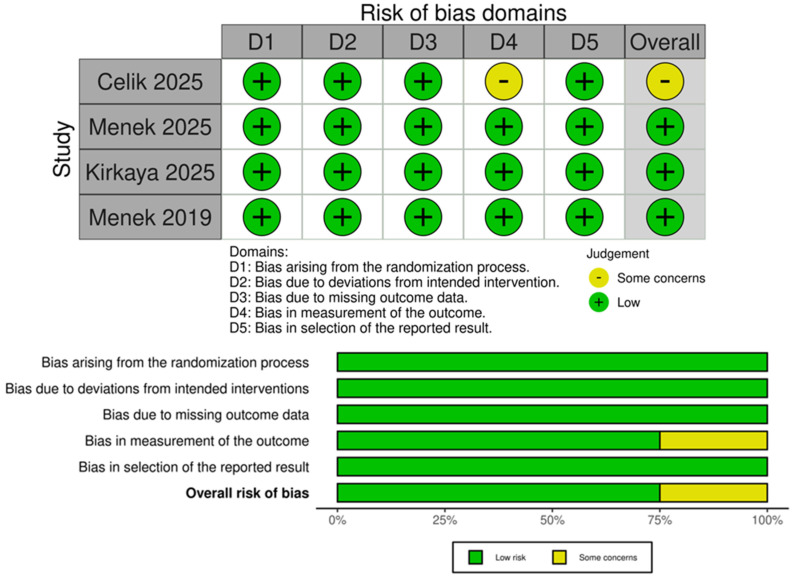
Risk of Bias Assessment (ROBINS-II) summary for the included studies. Among the four included studies, three were rated as having a low risk of bias, and one was rated as having some concerns. The most common source of bias was in the measurement of outcomes (Domain D4), with one study receiving a rating of “some concerns” [7,8,9,10].

Among the four included studies, three were rated as having a low risk of bias and one as having some concerns, according to the ROBINS-II tool. A detailed assessment is presented in Figure 8. The most frequent source of bias was related to the measurement of outcomes (Domain D4), with one study judged to have some concerns in this domain. Publication bias was assessed using contour-enhanced trim-and-fill funnel plots, which plot individual study weights against their effect estimates. While the funnel plots showed some asymmetry, interpretation is limited due to the small number of included studies (Figures S19–S24). According to the GRADE assessment, the quality of most outcomes were moderate (Table 3).

## 4. Discussion

This systematic review and meta-analysis of four randomized controlled trials (RCTs) involving 160 patients found that (1) Mulligan mobilization combined with conventional therapy significantly reduced pain at rest and during activity compared to conventional therapy alone; (2) it significantly improved functionality, range of motion, and joint position sense; and (3) no significant differences were found in quality of life (QoL) between the groups.

### 4.1. Principle Findings

The primary finding of this meta-analysis is the strong evidence (high certainty) supporting the addition of Mulligan mobilization to conventional therapy for improving multiple domains of patient outcomes, including pain, function, range of motion, and joint proprioception. The significant reductions in pain intensity at rest and, more substantially, during activity suggest a clinically meaningful benefit. The effect size for pain during activity is particularly noteworthy, indicating that the combined therapy effectively addresses movement-related pain—one of the main barriers to rehabilitation [5].

The superior improvement in functionality aligns with the reduction in pain, as decreased pain typically facilitates greater engagement in functional tasks [6]. Mulligan mobilizations, specifically Mobilization with Movement (MWM), aim to correct accessory joint positional faults, which can immediately restore pain-free movement and thereby enhance both function and range of motion [7]. The significant gains in range of motion are consistent with the mechanical mechanism of MWMs, where mobilization is applied simultaneously with active movement to overcome restrictions [7].

To improve interpretability, we additionally reported standardized effect sizes (Cohen’s d). These values demonstrated that Mulligan mobilization produced clinically meaningful improvements, with large effects for pain and functionality, range of motion, and joint position sense. Including effect sizes would help clinicians understand the magnitude of benefit beyond statistical significance alone.

### 4.2. Mechanistic Considerations

Furthermore, the improvement in joint position sense (MD-3.31) suggests a neurophysiological mechanism in which Mulligan techniques, through the stimulation of joint and periarticular mechanoreceptors within the joint capsule and periarticular tissues, enhance afferent feedback to the central nervous system and improve proprioceptive awareness and sensation, thereby improving coordinated movement and neuromuscular control [8]. The subgroup analysis for joint position sense, which showed a significant difference based on the risk of bias, indicates that study quality may influence this outcome, warranting cautious interpretation. Conversely, the absence of a significant difference in QoL is notable. Although pain, function, and mobility are important components of QoL, broader measures of QoL are often influenced by psychosocial factors not directly targeted by physiotherapy [9]. It is possible that treatment duration or instrument sensitivity was insufficient to detect a meaningful change in this outcome. The absence of QoL improvement may reflect that QoL measures encompass psychological and social dimensions beyond physical recovery, such as psychological well-being, fear-avoidance behavior, and patient expectations. Short intervention duration, small sample sizes, and use of generic QoL instruments (e.g., SF-36) may have limited sensitivity to detect meaningful changes. Furthermore, QoL measures may require a longer follow-up period than reported in the included studies to register a significant change.

### 4.3. Comparison with the Literature

Prior to this meta-analysis, the efficacy of Mulligan mobilization, particularly MWMs, was supported by several small-scale RCTs and narrative reviews focusing on individual joint dysfunctions such as knee osteoarthritis, shoulder impingement, and ankle sprains [10,11]. These studies commonly reported short-term benefits in pain and range of motion [8,9,10,11]. Manual therapy approaches, including MWM, have been widely applied in shoulder pathologies such as rotator cuff syndrome, subacromial impingement, and adhesive capsulitis, usually alongside exercise programs.

A prior systematic review on manual therapy in rotator cuff injuries found the evidence for manual therapy to be suggestive but inconclusive, especially when used as an adjunct to exercise [12]. The MWM approach, however, has been associated with significant improvements in pain, disability, and ROM in shoulder disorders. For example, Satpute et al. demonstrated that adding MWM significantly improved pain and abduction ROM in shoulder dysfunctions [13]. Similarly, an RCT by Menek et al. reported improvements in pain and quality of life in rotator cuff syndrome following Mulligan mobilization compared to conventional therapy [10].

Despite these findings, earlier trials were limited by small sample sizes, methodological heterogeneity, and inconsistent outcome reporting. Previous meta-analyses also combined heterogeneous shoulder pathologies, making it difficult to isolate the effect in rotator cuff disease specifically [12,13]. Consequently, uncertainty persisted regarding the magnitude and reliability of MWM’s benefits when added to conventional care.

### 4.4. Clinical Applications

The current meta-analysis strengthens this evidence base by providing pooled quantitative data derived exclusively from RCTs with high methodological quality. The significant improvement in joint position sense—a direct indicator of proprioceptive function—adds a new dimension to understanding the mechanism, extending beyond mechanical and pain-gating theories previously emphasized [8]. Our results for pain and functionality align with earlier literature, but the precision and statistical power gained through meta-analytic synthesis allow for stronger clinical recommendations. Earlier reviews [3,12,13] highlighted positive findings but emphasized the need for higher-quality evidence; this analysis addresses that gap by incorporating trials with predominantly low risk of bias.

The results strongly support integrating Mulligan mobilization into conventional rehabilitation programs for patients with musculoskeletal shoulder dysfunction. Given the high certainty of evidence across multiple outcomes, clinicians can be confident that adding MWM is likely to yield clinically meaningful improvements in pain, mobility, and function.

The improvement in range of motion and joint position sense is particularly important for patients returning to sport or performing high-demand tasks, as proprioceptive deficits are well-known risk factors for recurrent shoulder injury [13]. Therefore, MWMs should be considered not only as a pain-relief technique but also as a strategy for neuromuscular retraining and mechanical restoration. The lack of significant QoL improvement suggests that while MWMs produce substantial physical benefits, a comprehensive, patient-centered approach addressing psychosocial dimensions is necessary to optimize global well-being [9,23,24].

### 4.5. Limitations

Several limitations warrant consideration. Although all included trials were randomized, the total number of included studies (*n* = 4) and sample size (*n* = 160) remain relatively small, which limits the generalizability of our findings, particularly regarding QoL outcomes where heterogeneity was high (I^2^ = 76%). Second, despite an overall low risk of bias, substantial heterogeneity in outcomes such as pain during activity (I^2^ = 67%), joint position sense (I^2^ = 80%), and QoL (I^2^ = 76%) suggests variability in intervention protocols, patient characteristics, or outcome measures. The Baujat analysis identified specific studies, notably Menek et al. [10], as influential, suggesting that pooled results may be partially dependent on these trials. Third, the term ‘conventional therapy’ lacked a standardized definition across the included studies, typically encompassing a variable mix of therapeutic exercises, stretching, and modalities (e.g., heat/cold, TENS). This lack of protocol standardization in the comparator group is a source of clinical heterogeneity that warrants consideration in the interpretation of the pooled results [7]. Fourth, the unanimous geographic clustering of all included RCTs in Turkey is an important consideration. This concentration limits the external validity and generalizability of our findings, as patient populations, healthcare systems, and physiotherapy standards may differ significantly in other global regions.

### 4.6. Future Directions

Future research should aim to (1) conduct larger, multicenter RCTs with standardized MWM protocols to reduce heterogeneity and confirm these findings; (2) perform comparative effectiveness studies isolating the unique effects of MWM from other physiotherapy components; (3) include long-term follow-up to determine the durability of pain and function improvements; (4) explore the neurophysiological mechanisms underlying proprioceptive enhancement using advanced imaging or neurophysiological testing; and (5) assess QoL using condition-specific, validated instruments sensitive to short-term functional changes.

## 5. Conclusions

In patients with rotator cuff disease, adding Mulligan mobilization to conventional therapy improves pain, function, and proprioception. Routine integration in rehabilitation may enhance outcomes. Further studies with larger sample sizes and longer follow-up periods are warranted to validate and expand upon these findings.

## Figures and Tables

**Table 1:** Baseline characteristics of the included studies.

Study	Study Design	Country	No. Patients	Age *	Female, %	MG	CT	Affected Shoulder (L/R), %	Follow-up (Weeks)
Celik 2025 [9]	RCT	Turkey	30	MG: 43.5 CT: 51.1	MG: 20 CT: 60	15	15	MG: 47/53 CT: 47/53	3
Kirkaya 2025 [8]	RCT	Turkey	40	MG: 46.3 CT: 56.7	MG: 40 CT: 70	20	20	MG: 15/85 CT: 5/95	4
Menek 2025 [10]	RCT	Turkey	60	MG: 51.1 CT: 53.2	MG: 53 CT: 60	30	30	MG: 53/46 CT: 33/66	3
Menek 2019 [7]	RCT	Turkey	30	MG: 51.7 CT: 50.2	MG: 52 CT: 40	15	15	MG: 53/46 CT: 46/53	3

* mean.

**Table 2:** Study contributions to meta-analysis outcomes.

Study/Outcome	Pain (Rest)	Pain (Activity)	Functionality	ROM	QoL	JPS
Menek 2019 [7]	X	X	X	X	X	
Menek 2025 [10]	X	X	X	X		X
Celik 2025 [9]	X	X	X	X	X	X
Kirkaya 2025 [8]			X	X	X	

**Table 3:** Grade Assessment.

Certainty Assessment							№ of Patients		Effect		Certainty	Importance
№ of Studies	Study Design	Risk of Bias	Inconsistency	Indirectness	Imprecision	Other Considerations	Mulligan’s Technique with Conventional Therapy	Conventional Therapy Alone	Relative (95% CI)	Absolute (95% CI)		
**Rest pain intensity (follow-up: mean 3 weeks; assessed with: mean ± SD)**												
3	randomized trials	not serious	not serious	not serious	not serious	none	60	60	-	MD **1.19 lower** (1.64 lower to 0.74 lower)	⨁⨁⨁⨁ High	
**Active pain intensity (follow-up: mean 3 weeks; assessed with: mean ± SD)**												
3	randomized trials	not serious	not serious	not serious	not serious	none	60	60	-	MD **2.25 lower** (3.18 lower to 1.31 lower)	⨁⨁⨁⨁ High	
**Functionality (follow-up: mean 3 weeks; assessed with: mean ± SD)**												
4	randomized trials	not serious	not serious	not serious	not serious	none	80	80	-	MD **14.71 lower** (20.1 lower to 9.33 lower)	⨁⨁⨁⨁ High	
**Quality of Life (follow-up: mean 3 weeks; assessed with: mean ± SD)**												
3	randomized trials	not serious	not serious	not serious	serious ^a^	strong association	50	50	-	MD **10.58 higher** (3.18 lower to 24.34 higher)	⨁⨁⨁⨁ High ^a^	
**Range of Motion (follow-up: mean 3 weeks; assessed with: mean ± SD)**												
4	randomized trials	not serious	not serious	not serious	not serious	none	80	80	-	MD **19.82 higher** (11.25 higher to 28.39 higher)	⨁⨁⨁⨁ High	
**Joint position sense (follow-up: mean 3 weeks; assessed with: mean ± SD)**												
2	randomized trials	not serious	not serious	not serious	not serious	strong association	45	45	-	MD **3.31 lower** (6.22 lower to 0.4 lower)	⨁⨁⨁⨁ High	

^a^ significant heterogeneity.

## Data Availability

All data are available within the manuscript and its Supplementary Materials.

## Supplementary Materials

The following supporting information can be downloaded at: https://www.mdpi.com/article/10.3390/jcm14238352/s1, Table S1: Effect Sizes and Cohen’s Interpretation; Figure S1: LOO sensitivity analysis examining the robustness of the pooled effect size for greater pain intensity at rest; Figure S2: Baujat Plot identifying the contribution of individual studies to the overall heterogeneity and effect size; Figure S3: LOO Sensitivity Analysis for the effect of the combined therapy on pain intensity during activity; Figure S4: Baujat Plot illustrating the influence of individual studies on the meta-analysis; Figure S5: LOO Sensitivity Analysis testing the robustness of the pooled effect for patient functionality; Figure S6: Baujat Plot illustrating the influence of individual studies on the meta-analysis; Figure S7: LOO Sensitivity Analysis testing the robustness of the pooled effect for range of motion; Figure S8: Baujat Plot illustrating the influence of individual studies on the meta-analysis for range of motion; Figure S9: LOO Sensitivity Analysis testing the robustness of the pooled effect for joint position sense (JPS); Figure S10: Baujat Plot illustrating the influence of individual studies on the meta-analysis for joint position sense; Figure S11: LOO Sensitivity Analysis testing the robustness of the pooled effect for QoL; Figure S12: Baujat Plot illustrating the influence of individual studies on the meta-analysis for QoL; Figure S13: Forest plot illustrating the risk of bias subgroup analysis for pain intensity at rest; Figure S14: Forest plot illustrating the risk of bias subgroup analysis for pain intensity during activity; Figure S15: Forest plot illustrating the risk of bias subgroup analysis for patient functionality; Figure S16: Forest plot illustrating the risk of bias subgroup analysis for range of motion; Figure S17: Forest plot illustrating the risk of bias subgroup analysis for joint position sense; Figure S18: Forest plot illustrating the risk of bias subgroup analysis for QoL; Figure S19: Contour-Enhanced Trim-and-Fill Funnel Plot for pain intensity at rest; Figure S20: Contour-Enhanced Trim-and-Fill Funnel Plot for pain intensity during activity; Figure S21: Contour-Enhanced Trim-and-Fill Funnel Plot for functionality; Figure S22: Contour-Enhanced Trim-and-Fill Funnel Plot for range of motion; Figure S23: Contour-Enhanced Trim-and-Fill Funnel Plot for joint position sensation; Figure S24: Contour-Enhanced Trim-and-Fill Funnel Plot for QoL.
