# Supplementary material for: Mulligan Mobilization Combined with Conventional Therapy vs. Conventional Care Alone in Patients with Rotator Cuff Disease: A Systematic Review and Meta-Analysis of Randomized Controlled Trials
Source: J Clin Med. 2025 Nov 24;14(23):8352. doi: 10.3390/jcm14238352 (PMC12692990; doi:10.3390/jcm14238352)
Supplement: Supplementary file 1 [file jcm-14-08352-s001.zip › jcm-3960895-supplementary.pdf]

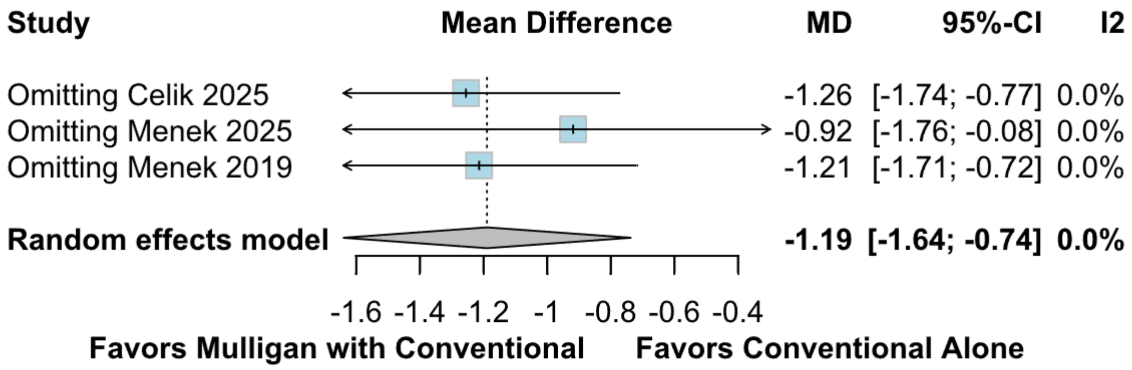

Figure S1. LOO sensitivity analysis examining the robustness of the pooled effect size for greater pain intensity at rest. The analysis shows that the overall effect size remained consistent and statistically significant across all iterations, confirming the stability of the primary result.

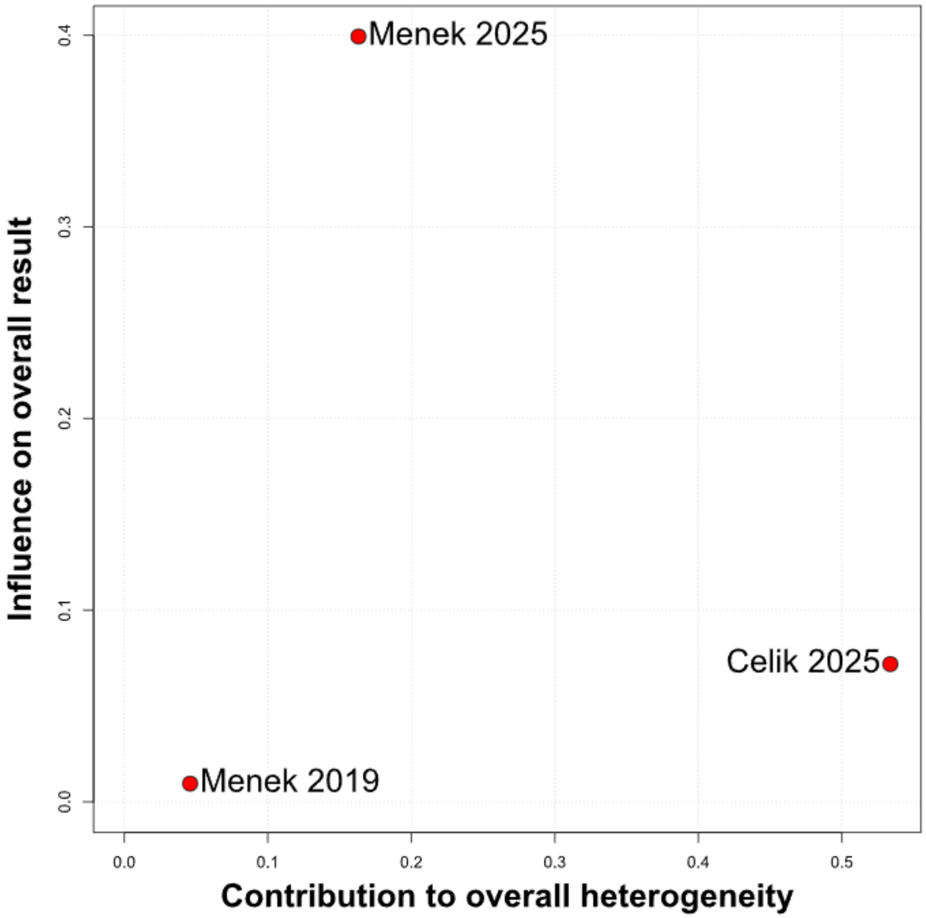

Figure S2. Baujat Plot identifying the contribution of individual studies to the overall heterogeneity and effect size. The plot highlights the study by Menek et al. (2025) as potentially influential, contributing substantially to the overall result and heterogeneity of the meta-analysis.

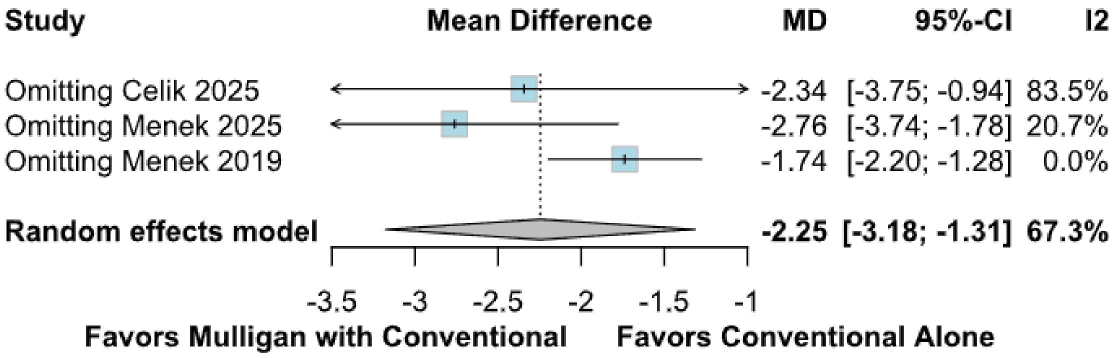

Figure S3. LOO Sensitivity Analysis for the effect of the combined therapy on pain intensity during activity. The analysis demonstrates robustness, as the overall effect size remained consistent and significant across all iterations. This confirms that no single study disproportionately influenced the overall outcome.

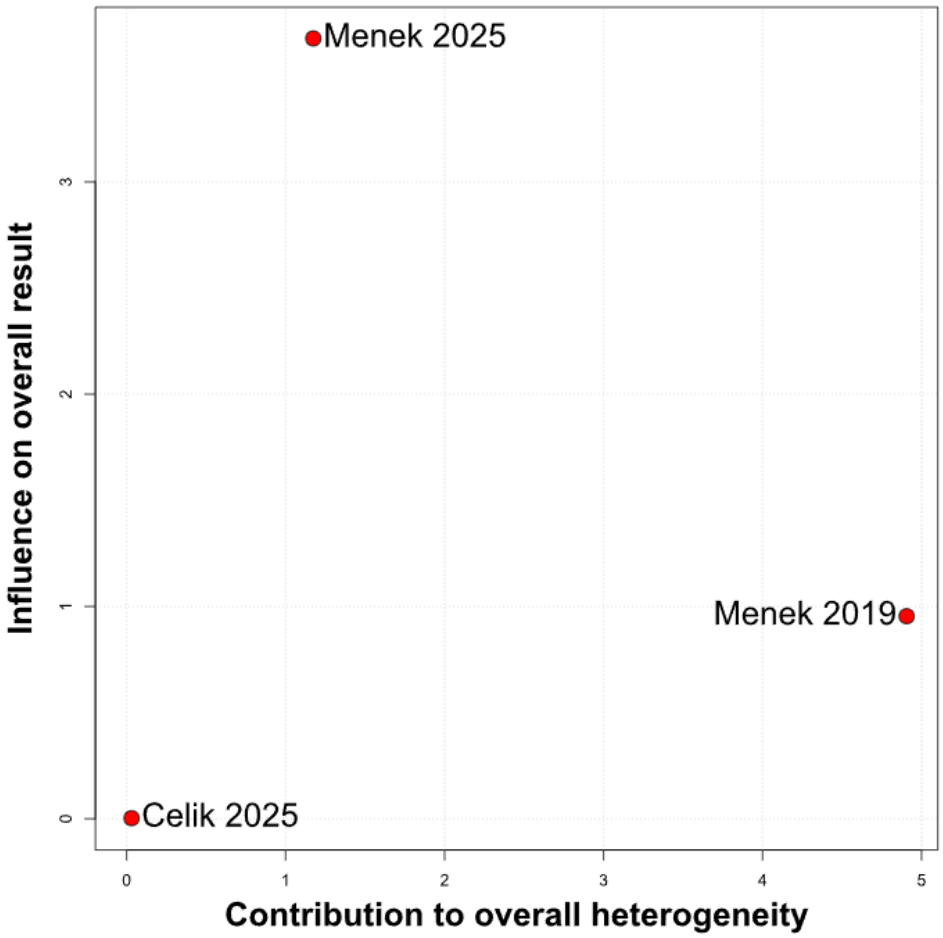

Figure S4. Baujat Plot illustrating the influence of individual studies on the meta-analysis. The plot identifies Menek et al. (2025) as potentially influential and contributing substantially to the overall effect size. Additionally, the plot highlights Menek (2019) as contributing substantially to the overall heterogeneity.

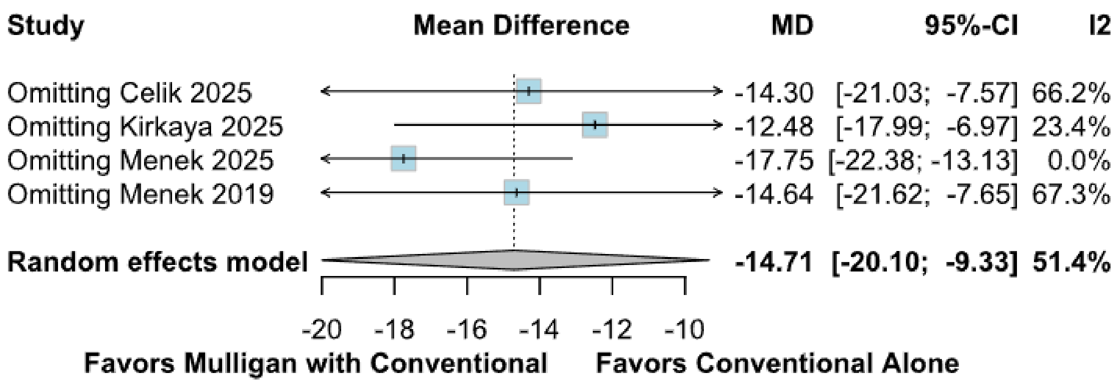

Figure S5. LOO Sensitivity Analysis testing the robustness of the pooled effect for patient functionality. The analysis shows the overall effect size remained consistent and statistically significant across all iterations confirming the stability of the result.

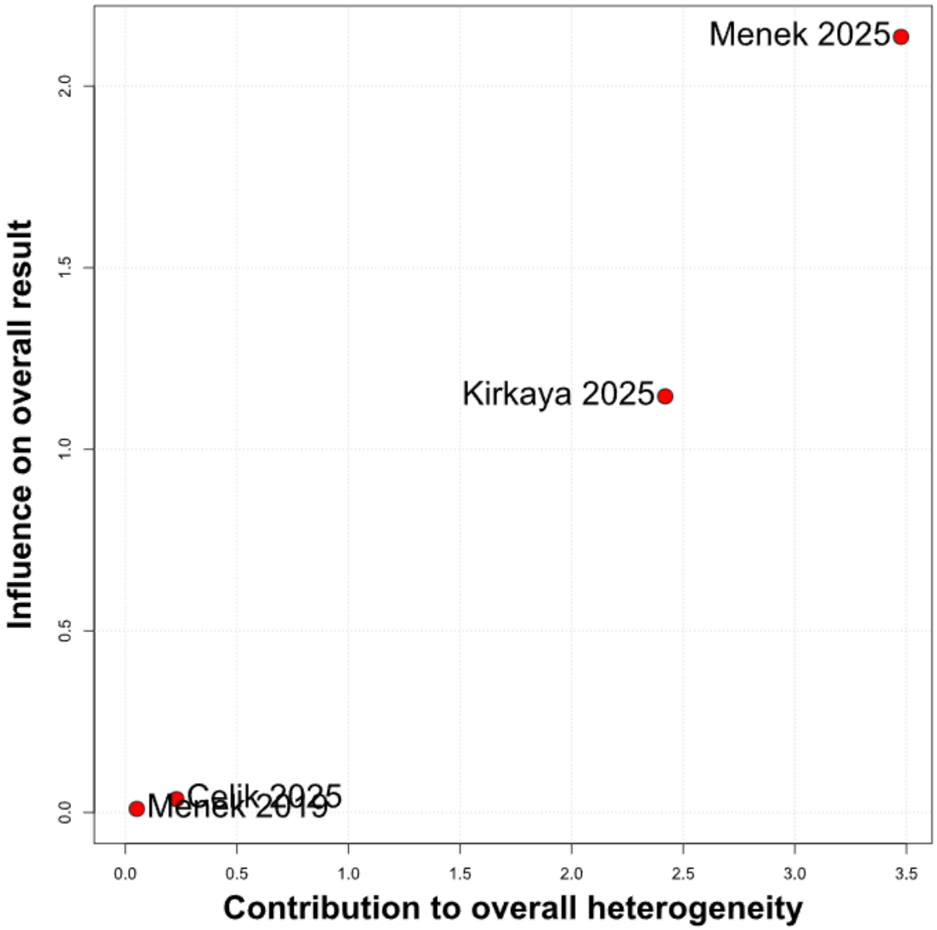

Figure S6. Baujat Plot illustrating the influence of individual studies on the meta-analysis. The plot identifies Menek et al. (2025) and Kirkaya et al. (2025) as potentially influential studies, contributing substantially to both the overall effect size and the heterogeneity of the meta-analysis.

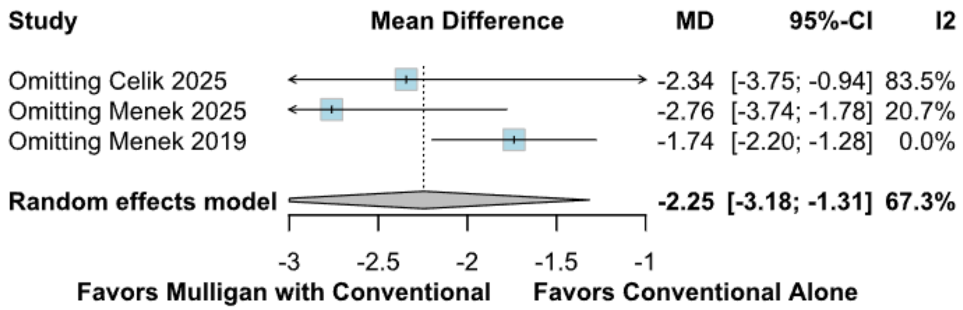

Figure S7. LOO Sensitivity Analysis testing the robustness of the pooled effect for range of motion. The analysis demonstrates that the overall effect size remained consistent and statistically significant across all iterations confirming the stability of the meta-analysis result.

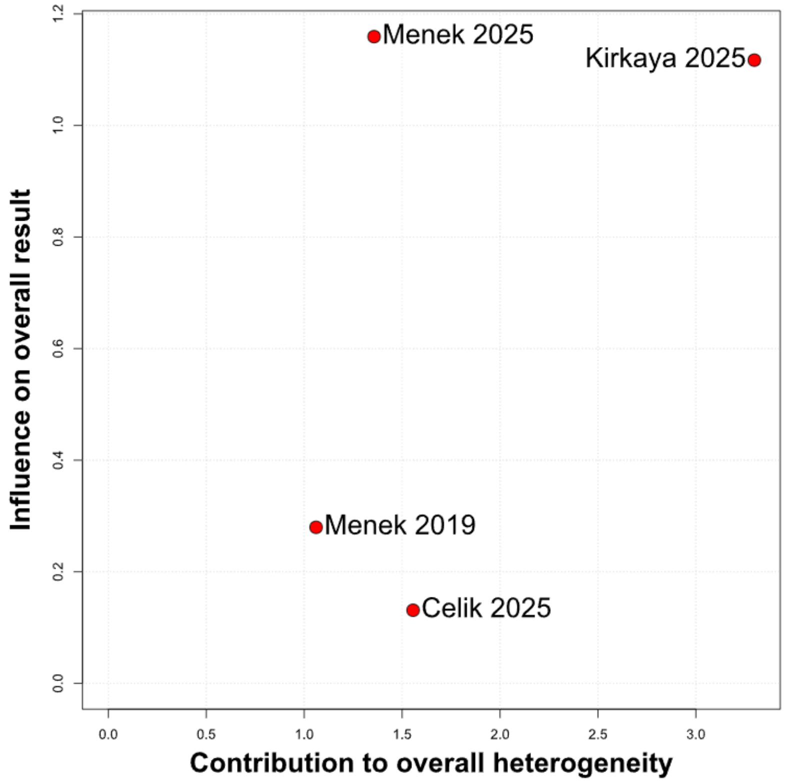

Figure S8. Baujat Plot illustrating the influence of individual studies on the meta-analysis for range of motion. The plot identifies Menek et al. (2025) and Kirkaya et al. (2025) as potentially influential studies, contributing substantially to both the overall effect size and the heterogeneity of the meta-analysis.

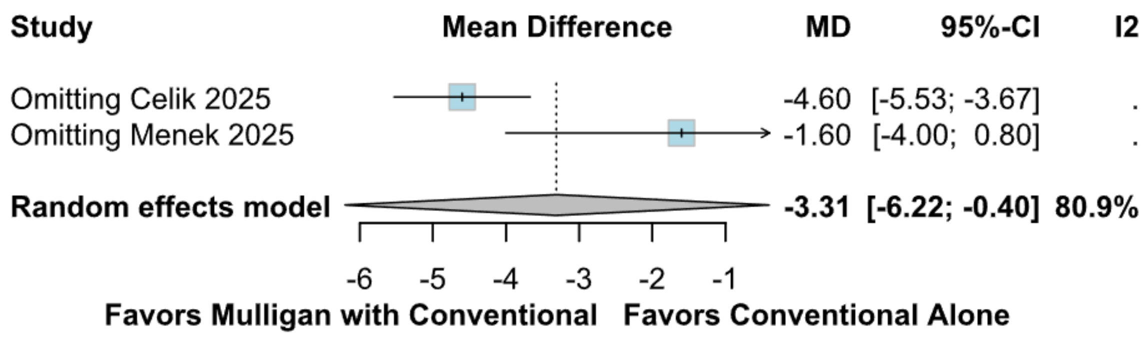

Figure S9. LOO Sensitivity Analysis testing the robustness of the pooled effect for joint position sense (JPS). The analysis confirms the stability of the result, as the overall effect size remained consistent and statistically significant across all iterations. This indicates that no single study had a disproportional influence on the overall outcome.

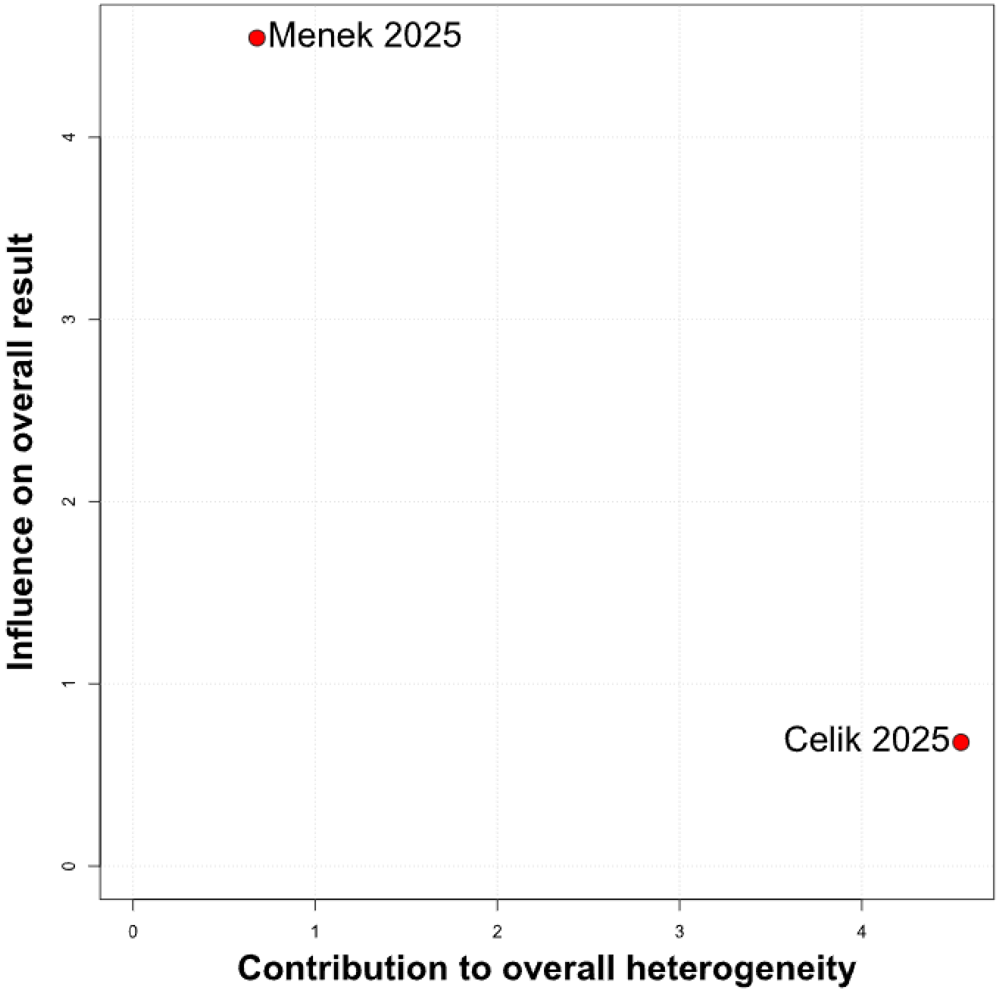

Figure S10. Baujat Plot illustrating the influence of individual studies on the meta-analysis for joint position sense. The plot identifies Menek et al. (2025) as potentially influential, contributing substantially to the overall effect size. Additionally, the plot highlights Celik (2025) as contributing substantially to the overall heterogeneity.

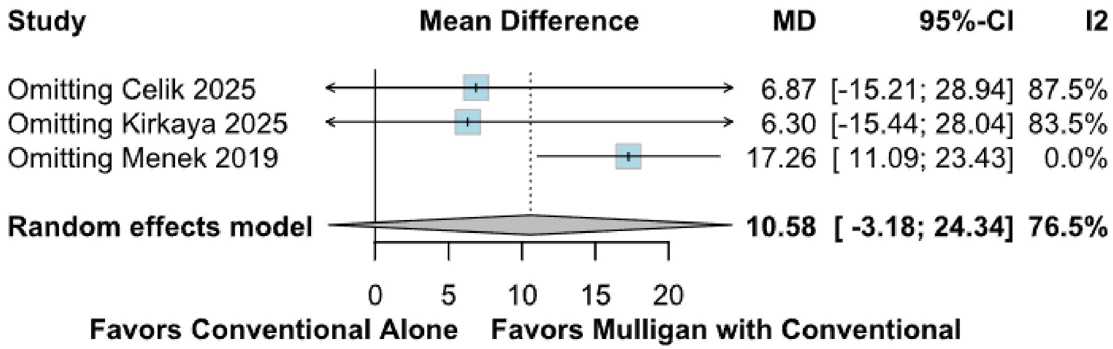

Figure S11. LOO Sensitivity Analysis testing the robustness of the pooled effect for QoL. The analysis confirms the stability of the result: the overall effect size remained consistent and non-significant across all iterations suggesting no single study had a disproportional influence on the overall outcome.

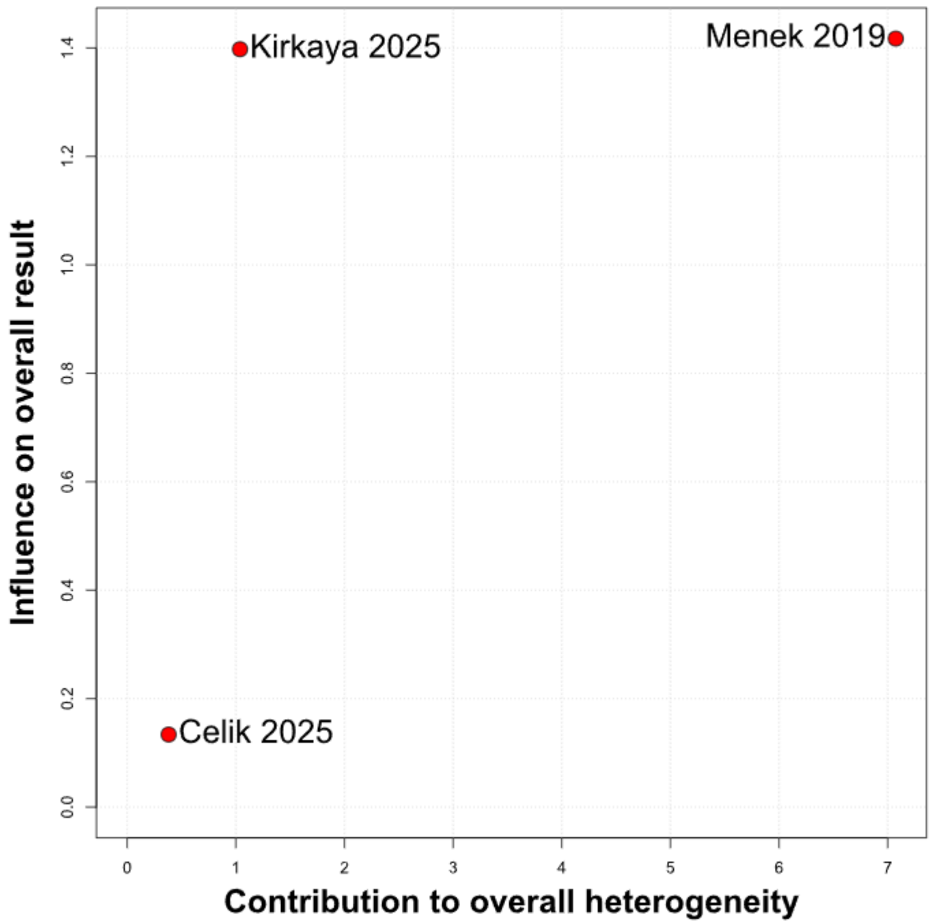

Figure S12. Baujat Plot illustrating the influence of individual studies on the meta-analysis for QoL. The plot identifies Kirkaya et al. (2025) as potentially influential, contributing substantially to the overall effect size. Additionally, the plot highlights Menek (2019) as contributing substantially to the overall heterogeneity.

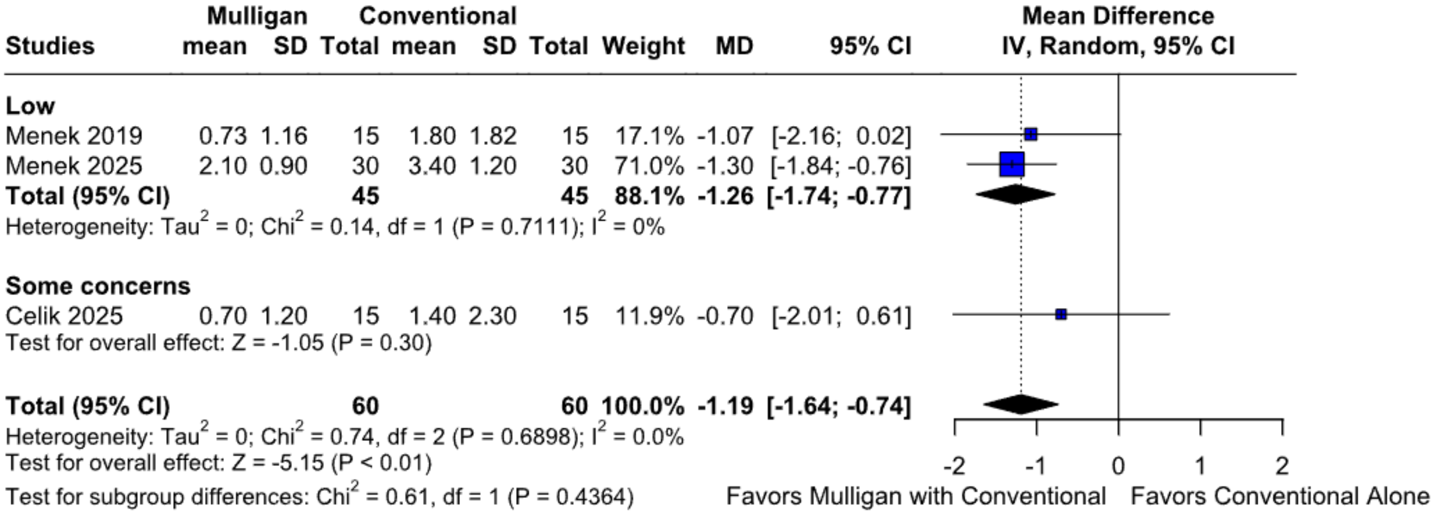

Figure S13. Forest plot illustrating the risk of bias subgroup analysis for pain intensity at rest. No statistically significant differences were observed between the subgroups, confirming that the risk of bias did not significantly affect the overall outcome. Heterogeneity remained low across the subgroup.

Studies	Mulligan mean	Mulligan SD	Mulligan Total	Conventional mean	Conventional SD	Conventional Total	Weight	MD	95% CI
**Low**									
Menek 2019	2.46	1.72	15	5.60	1.12	15	31.7%	-3.14	[-4.18; -2.10]
Menek 2025	3.20	1.00	30	4.90	0.90	30	45.9%	-1.70	[-2.18; -1.22]
**Total (95% CI)**			**45**			**45**	**77.6%**	**-2.34**	**[-3.75; -0.94]**
Heterogeneity: $Tau^2 = 0.8662$; $Chi^2 = 6.08$, df = 1 (P = 0.0137); $I^2 = 83.5\%$									
**Some concerns**									
Celik 2025	3.40	1.70	15	5.50	2.40	15	22.4%	-2.10	[-3.59; -0.61]
Test for overall effect: Z = -2.77 (P < 0.01)									
**Total (95% CI)**			**60**			**60**	**100.0%**	**-2.25**	**[-3.18; -1.31]**
Heterogeneity: $Tau^2 = 0.4315$; $Chi^2 = 6.11$, df = 2 (P = 0.0471); $I^2 = 67.3\%$									
Test for overall effect: Z = -4.73 (P < 0.01)									
Test for subgroup differences: $Chi^2 = 0.05$, df = 1 (P = 0.8156)									

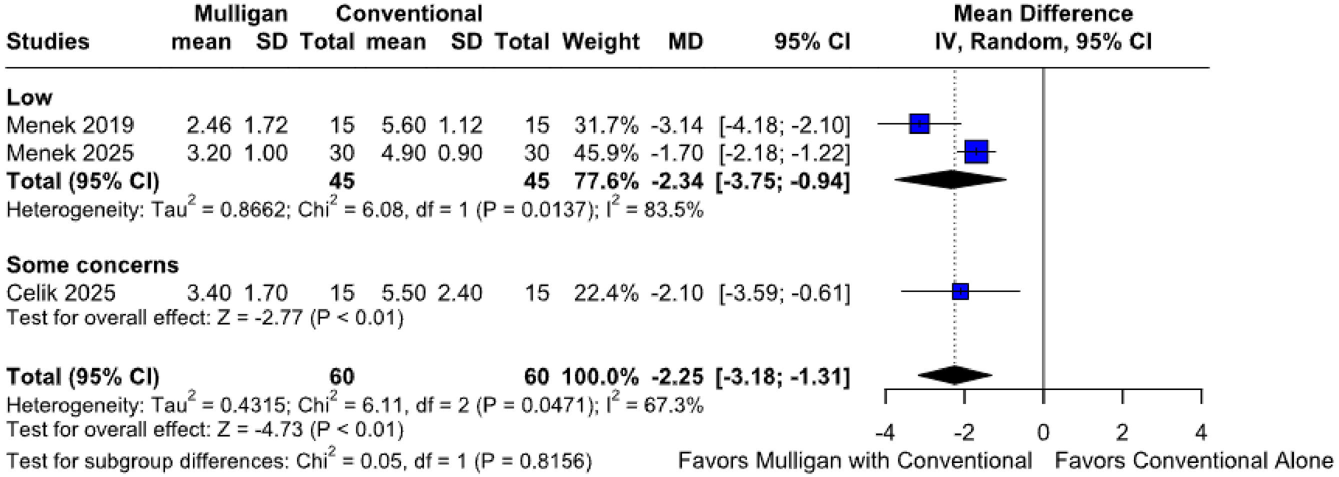

Figure S14. Forest plot illustrating the risk of bias subgroup analysis for pain intensity during activity. No statistically significant differences were observed between the subgroups, indicating that the risk of bias did not significantly modify the overall effect. Moderate heterogeneity was maintained.

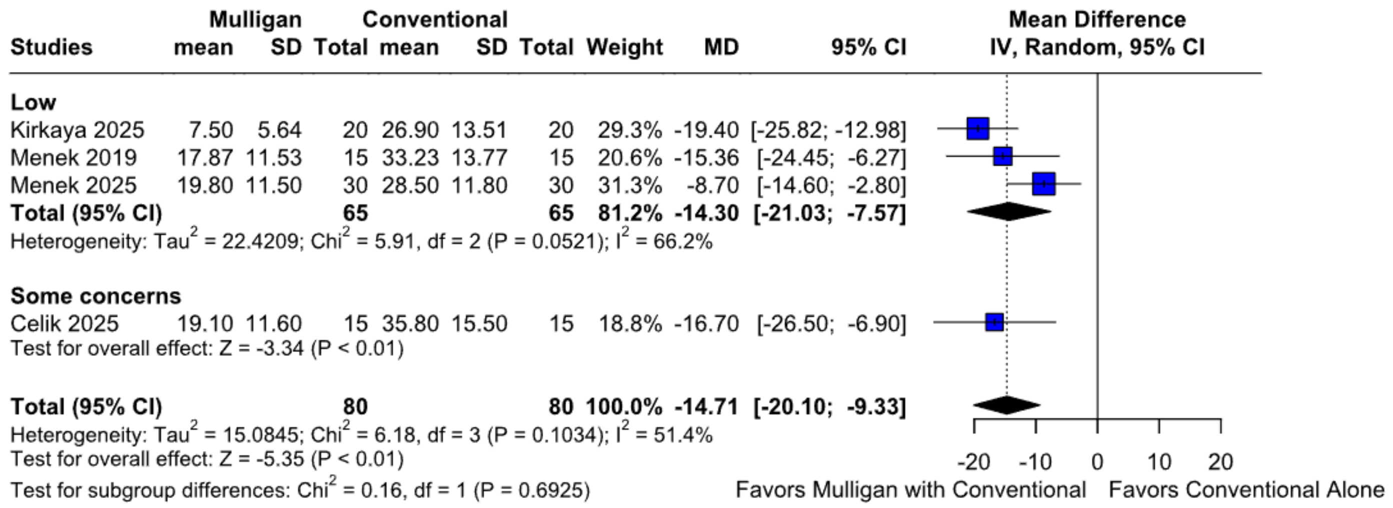

Figure S15. Forest plot illustrating the risk of bias subgroup analysis for patient functionality. No statistically significant differences were observed between the subgroups, indicating that the risk of bias did not significantly affect the overall outcome. Moderate heterogeneity was maintained.

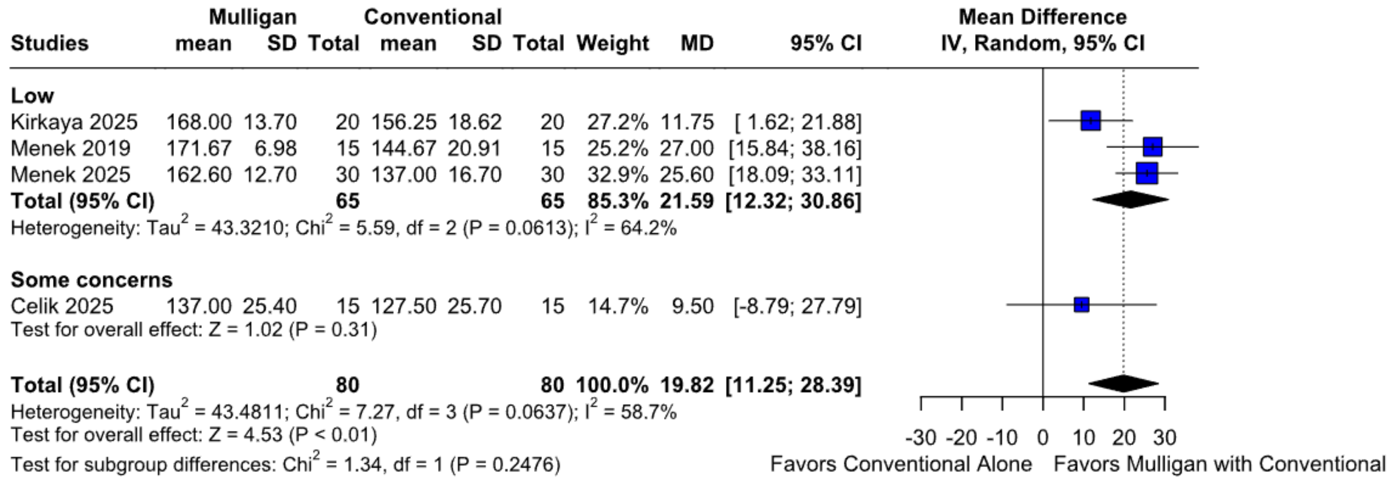

Figure S16. Forest plot illustrating the risk of bias subgroup analysis for range of motion. No statistically significant differences were observed between the subgroups, suggesting that the risk of bias did not significantly affect the overall outcome. Moderate heterogeneity was maintained.

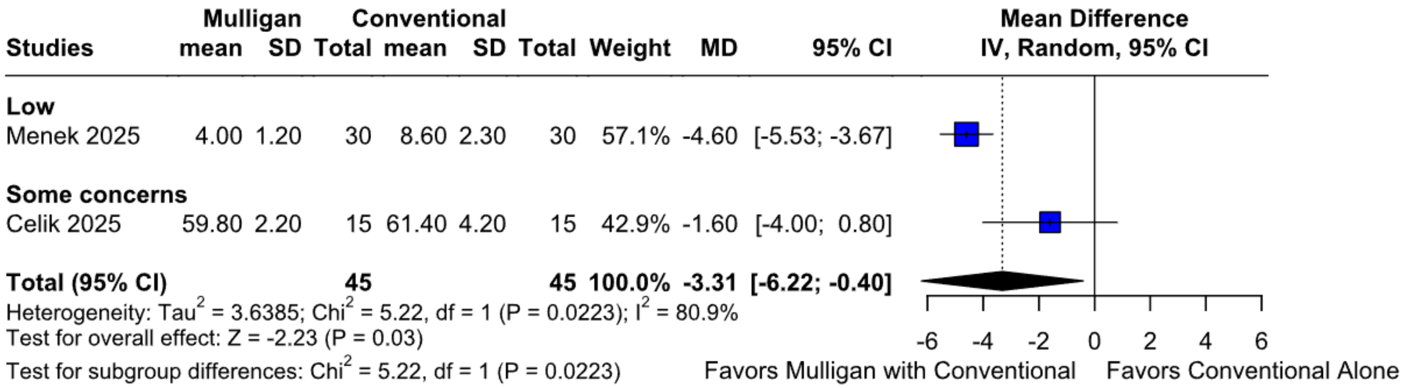

Figure S17. Forest plot illustrating the risk of bias subgroup analysis for joint position sense. A statistically significant difference was observed between the subgroups, suggesting that the risk of bias significantly influences the overall effect. High heterogeneity was maintained.

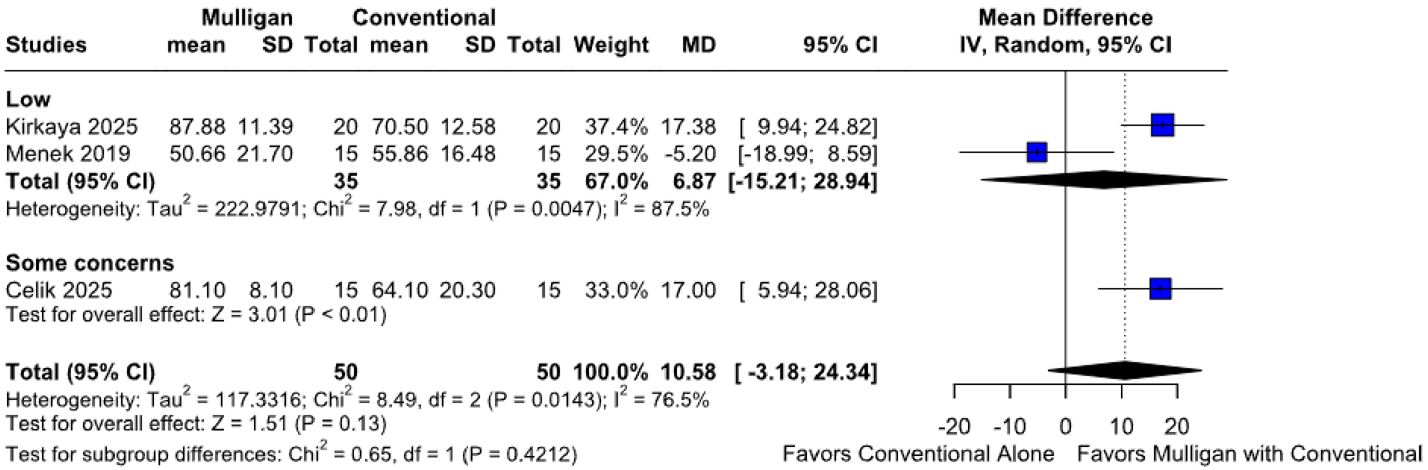

Figure S18. Forest plot illustrating the risk of bias subgroup analysis for QoL. No statistically significant differences were observed between the subgroups , suggesting that the risk of bias did not significantly affect the overall outcome for QoL. High heterogeneity was maintained.

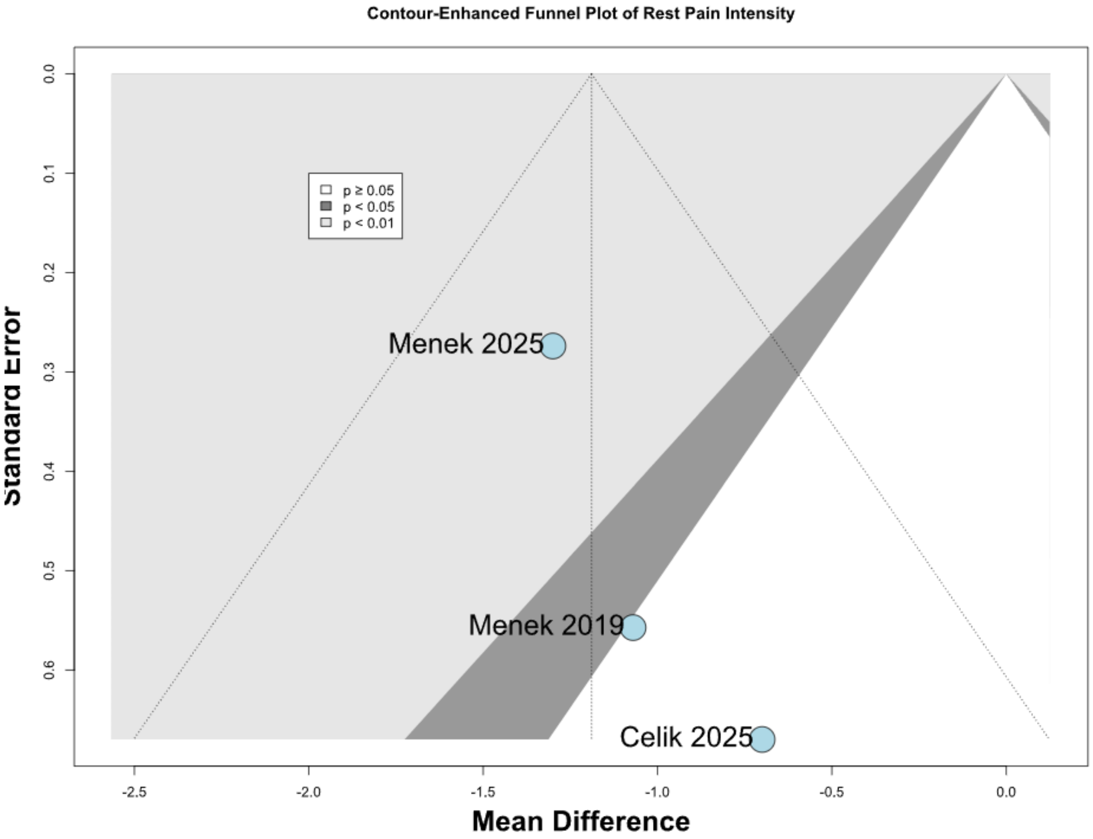

Figure S19. Contour-Enhanced Trim-and-Fill Funnel Plot for pain intensity at rest. The plot illustrates individual study weights against point estimates.

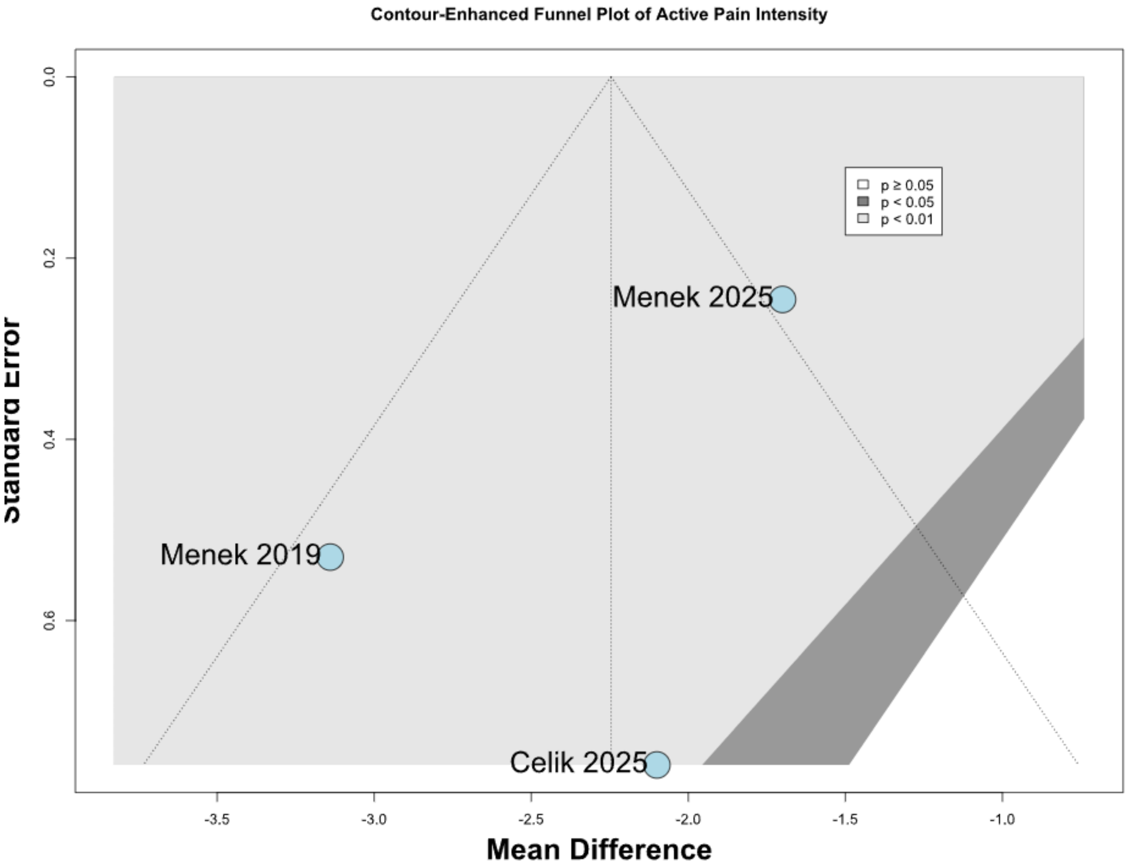

Figure S20. Contour-Enhanced Trim-and-Fill Funnel Plot for pain intensity during activity. The plot illustrates individual study weights against point estimates.

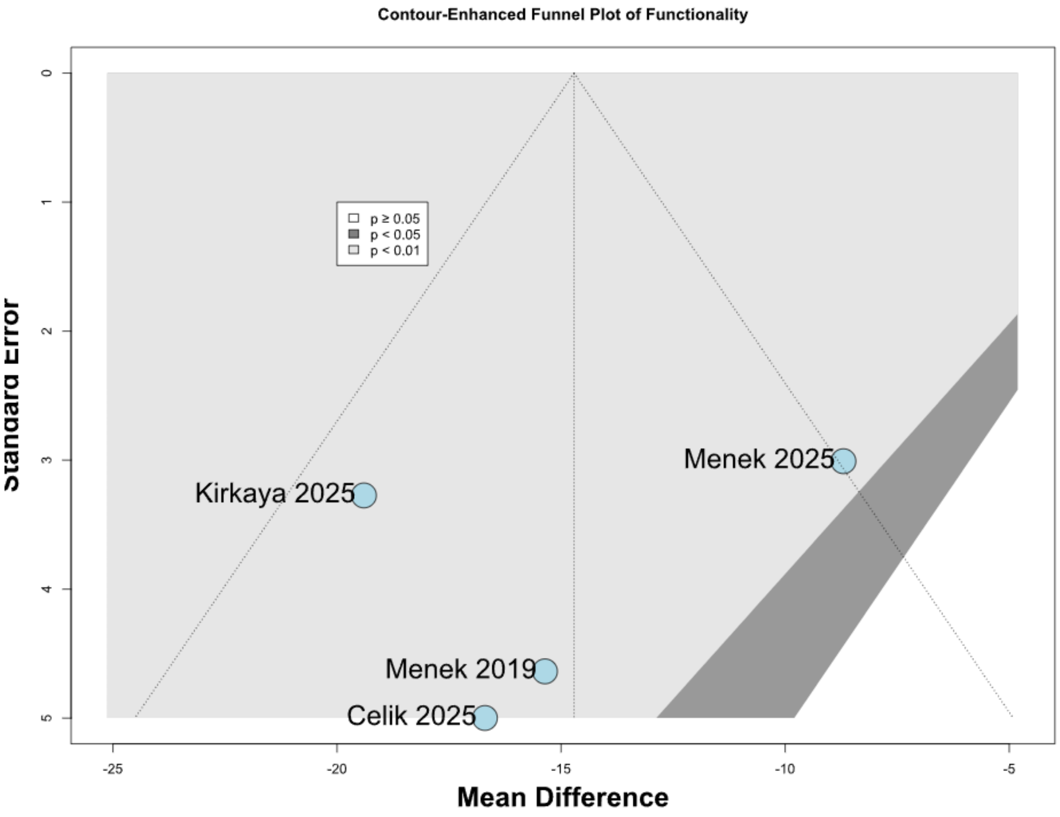

Figure S21. Contour-Enhanced Trim-and-Fill Funnel Plot for functionality. The plot illustrates individual study weights against point estimates.

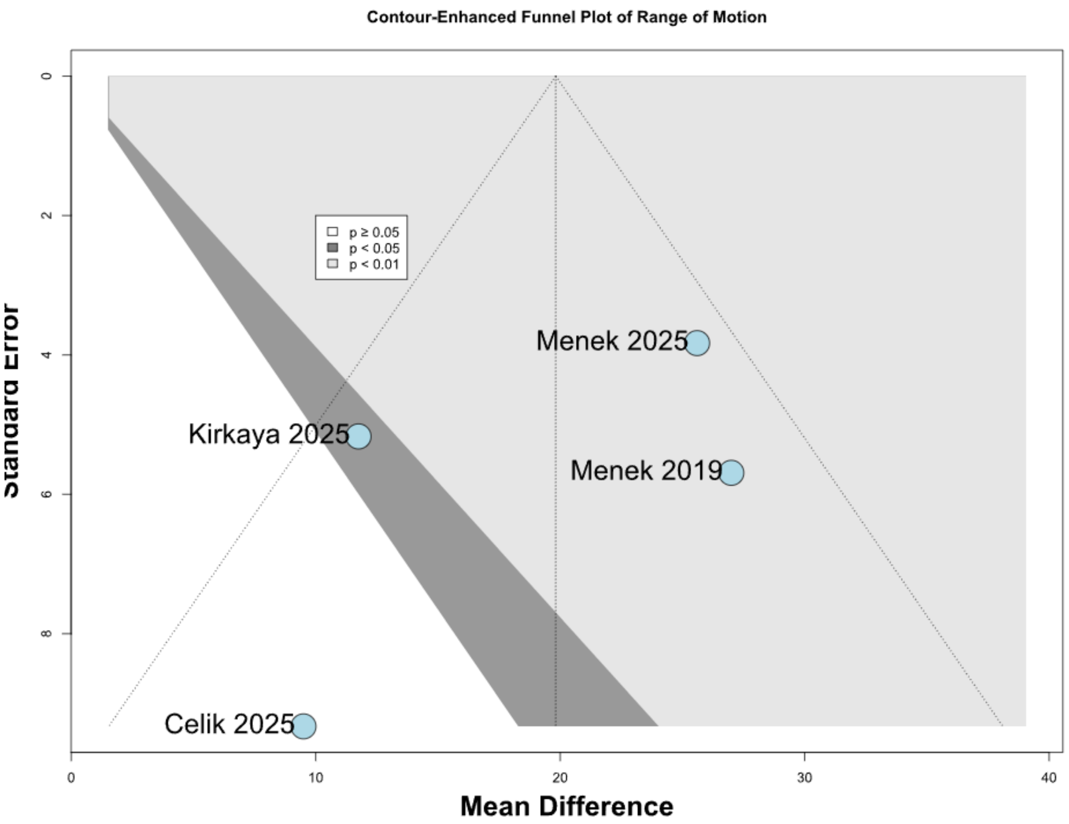

Figure S22. Contour-Enhanced Trim-and-Fill Funnel Plot for range of motion. The plot illustrates individual study weights against point estimates.

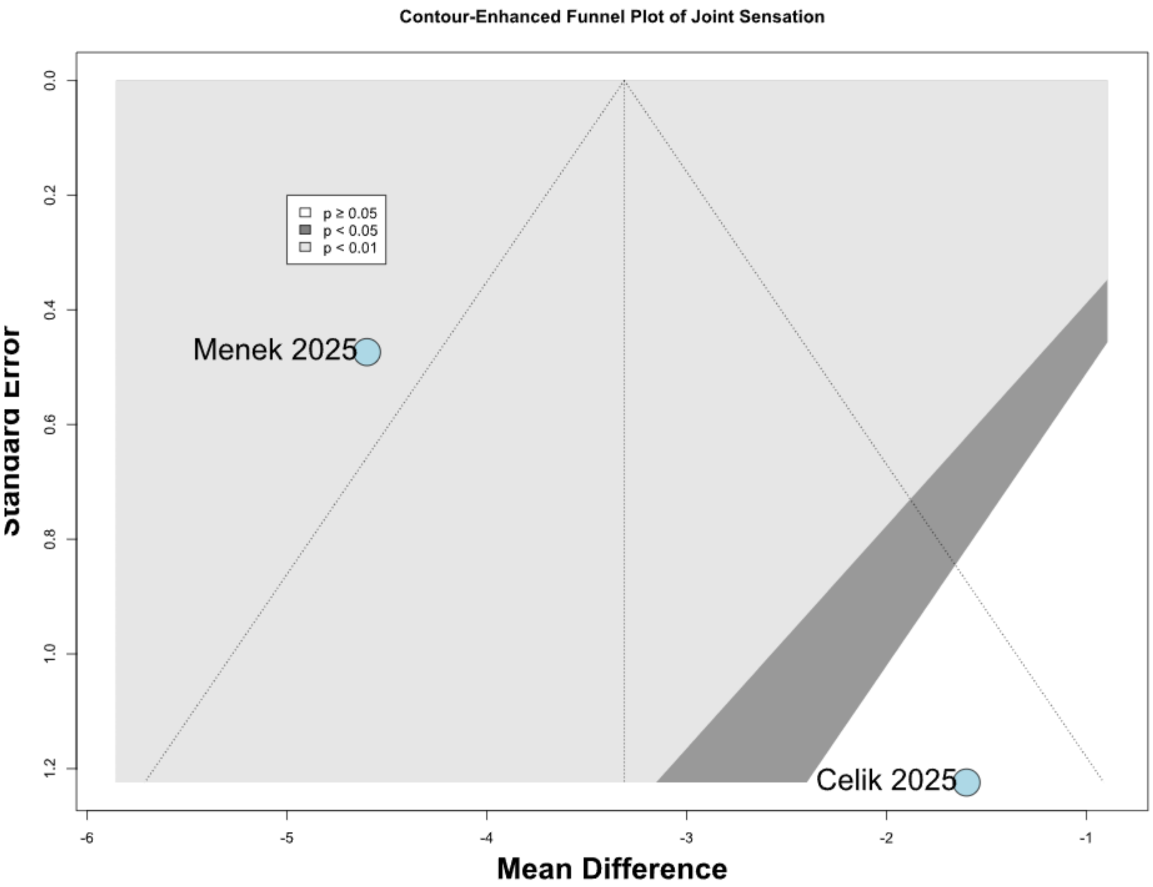

Figure S23. Contour-Enhanced Trim-and-Fill Funnel Plot for joint position sensation. The plot illustrates individual study weights against point estimates.

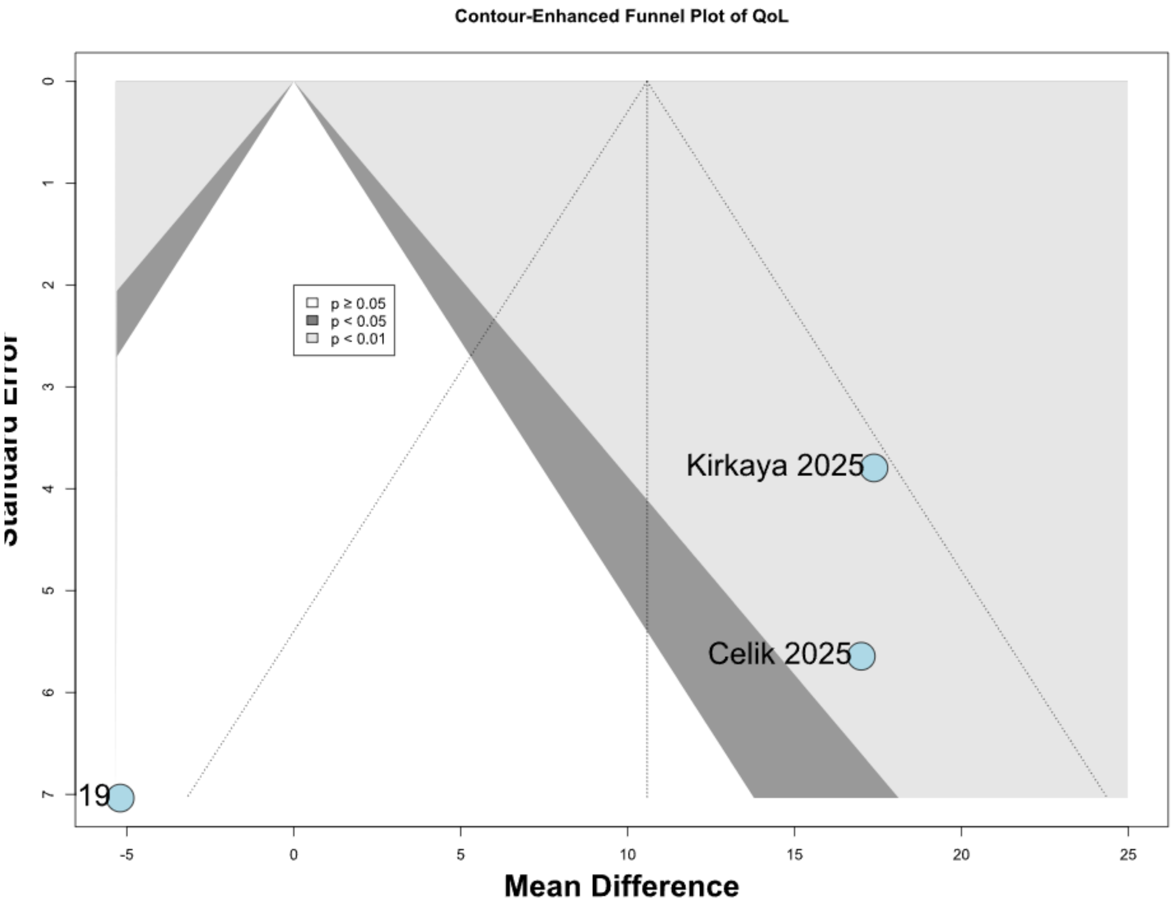

Figure S24. Contour-Enhanced Trim-and-Fill Funnel Plot for QoL. The plot illustrates individual study weights against point estimates.

Supplementary Table S1: Effect Sizes and Cohen’s Interpretation

Outcome	Mean Difference (MD)	Cohen’s d	Effect Size Interpretation
Pain intensity at rest	-1.19	-0.80	**Large effect**
Pain intensity during activity	-2.25	-1.60	**Large effect**
Functionality	-14.71	-1.24	**Large effect**
Range of motion	+19.82	+1.11	**Large effect**
Joint position sense	-3.31	-1.47	**Large effect**
